# Characteristics and outcomes of e-cigarette exposure incidents reported to 10 European Poison Centers: a retrospective data analysis

**DOI:** 10.1186/s12971-017-0141-z

**Published:** 2017-08-16

**Authors:** Constantine I. Vardavas, Charis Girvalaki, Filippos T Filippidis, Mare Oder, Ruth Kastanje, Irma de Vries, Lies Scholtens, Anita Annas, Silvia Plackova, Rajka Turk, Laima Gruzdyte, Fátima Rato, Dieter Genser, Helmut Schiel, Andrea Balázs, Elaine Donohoe, Alexander I. Vardavas, Manolis N. Tzatzarakis, Aristidis M. Tsatsakis, Panagiotis K. Behrakis

**Affiliations:** 1 Biomedical Research Foundation of the Academy of Athens (BRFAAA), Athens, Greece; 2 Institute of Public Health, American College of Greece, Athens, Greece; 3 Department of Primary Care and Public Health, School of Public Health, Imperial College London, London, UK; 4 Poisoning Information Center, Estonian Health Board, Tallinn, Estonia; 5 Dutch Poisons Information Center, University Medical Center Utrecht, Utrech, The Netherlands; 6 Swedish Poisons Information Center, SE-171 76 Stockholm, Sweden; 7 National Toxicological Information Center, University Hospital Bratislava, Bratislava, Slovakia; 8 Institute for Medical Research an Occupational Health, Poison Control Center, Zagreb, Croatia; 9 Poison Information Bureau, Health emergency situations center of the Ministry of health, Vilnius, Lithuania; 10 National Institute Of Medical Emergency, Lisboa, Portugal; 11 Poisons Information Center, Vienna, Austria; 12 National Public Health Center, National Directorate of Chemical Safety, Health Toxicological Information Service, Budapestᅟ, Hungary; 13 National Poisons Information Center, Beaumont Hospital, Dublin, Ireland; 14 Laboratory of Toxicology, Medical School, University of Crete, Voutes, 71409 Heraklion, Crete Greece

**Keywords:** Poisoning, e-liquid, Poison centers, Europe

## Abstract

**Background:**

The use of e-cigarettes has increased during the past few years. Exposure to e-cigarette liquids, whether intentional or accidental, may lead to adverse events our aim was to assess factors associated with e-cigarette exposures across European Union Member States (EU MS).

**Methods:**

A retrospective analysis of exposures associated with e-cigarettes reported to national poison centers was performed covering incidents from 2012 to March 2015 from 10 EU MS. De-identified and anonymous raw data was acquired.

**Results:**

In total, 277 incidents were reported. Unintentional exposure was the most frequently cited type of exposure (71.3%), while e-cigarette refill vials were responsible for the majority of the reported incidents (87.3%). Two-thirds of all exposures (67.5%) occurred as ingestion of e-liquids, which was more frequent among children (≤ 5 years, 6–18 years) compared to adults (87.0% vs. 59.3% vs. 57.6%, *p* < 0.001 respectively), exposure via the respiratory (5.4% vs. 22.2% vs. 22.2%, *p* < 0.001) were more frequent among paediatric patients while ocular routes (2.2% vs. 3.7% vs. 11.4%, *p* = 0.021) were more frequent among adults. Logistic regression analyses indicated that paediatric incidents (≤ 5 years) were more likely to be through ingestion (adjusted Odds Ratio [aOR] = 4.36, 95% Confidence Interval [C.I.]: 1.87–10.18), but less likely to have a reported clinical effect (aOR = 0.41, 95% C.I.: 0.21–0.82).

**Conclusions:**

Our study highlighted parameters related to e-cigarette exposure incidents in 10 EU MS, the results of which indicate that consideration should be given to the design features which may mitigate risks, thereby protecting users, non-users and especially children.

## Background

The European Tobacco Products Directive (TPD) 2014/40/EU, regulates e-cigarettes across 28 European Member States (EU MS) and includes provisions to harmonise the safety and technical design specifications for e-cigarettes and their refill containers [1]. The TPD and its accompanying implementing acts regulate many aspects of e-cigarette design including but not limited to the volume of the refill container, the nicotine and ingredient content and tamper resistant and child-resistant refill containers [2]. In the USA, the food and Drug administration (FDA) in 2016 also finalized a deeming rule covering the manufacture, import, packaging, labelling, advertising, promotion, sale, and distribution of Electronic Nicotine Delivery Systems (ENDS) in the United States [3].

E-cigarette refill liquids contain nicotine and a wide variety of chemical components such as humectants, flavours, and potential impurities [4, 5]. Exposure to such liquids, whether intentional or accidental, may lead to adverse events [6]. Indeed, several recent reports from poison centers, primarily from the United States (US) and the United Kingdom (UK), have revealed a significant increase in the number of reported incidents of poisoning with e-cigarette liquids and associations with specific demographic characteristics [4, 5]. However, data from across Europe, are only sporadically available, mostly in the form of case reports in the literature [7–9].

Due to the fact that the e-cigarette market and respective experimentation or use has been documented to increase over the past few years in Europe [10, 11], it is of significant interest to evaluate the current landscape with regards to adverse exposures to e-cigarette liquids, especially in light of the recent adoption of technical design parameters that e-cigarettes will need to adhere to within the context of alignment with the provisions of the European TPD.

In light of the above our aim was to assess the factors associated with e-cigarette exposure incidents reported to poison centers across multiple EU MS.

## Methods

### Data collection

A retrospective analysis of exposures associated with e-cigarettes as reported to national poison centers was conducted, within the context of a study prepared for the Directorate General for Health and Food Safety (EU DG Health and Food Safety), (Chafea/2014/Health/17).

A request for raw data was sent to poison centers within the 22 EU MS. Ten poison centers from equal EU MS (Sweden, The Netherlands, Ireland, Portugal, Austria, Slovakia, Lithuania, Hungary, Croatia and Estonia) agreed to provide raw data covering incidents from 2012 to March 2015. However, three EU MS (Ireland-53 out of 90 incidents, Croatia-3 incidents and Estonia- 10 incidents) were only able to provide summary statistics for the specific incidents reported to their centers, due to lack of detailed data or confidentiality regulations. These 66 incidents were excluded from the statistical analysis and hence all statistical analyses were performed on the 277 incidents from the remaining eight EU MS (Sweden, Ireland-37 out of 90 incidents, The Netherlands, Portugal, Austria, Slovakia, Lithuania and Hungary).

All information requested and received was de-identified and anonymous. Data on age (Paediatric incidents: ≤5 years, 6–18 years, adults: ≥19 years); gender (male, female); route of exposure (ingestion, respiratory, dermal and ocular); initial type of exposure (e-cigarette refill liquid, e-cigarette non refillable, unknown); management of incident (residence/on site, hospital, ambulance, other/unknown); reported clinical outcome (minor effects, moderate effects, major effects, death); and reason of exposure (intentional, unintentional, abuse, misuse, suspected suicide or unknown reason) were collected from the archive of each poison center. For the purpose of our analyses intentional, abuse and misuse were grouped together as intentional exposures.

### Statistical analysis

All variables are displayed as frequencies and percentages (n, %), unless stated otherwise. Chi-square tests were used for associations between categorical variables. Multivariable binary logistic regression models were performed to investigate the association of having an effect from poisoning and route of exposure with age, gender and exposure characteristics. Level of significance was set at *p* < 0.05 and regression results are presented as adjusted Odds Ratios (aOR) with 95% Confidence Intervals (CI). Data were analyzed using the SPSS statistical package version 21.0 (SPSS, Chicago, IL).

## Results

### Incidents, geographical distribution and time trends

Data for a total of 343 incidents were initially reported while 277 were analysed. Sweden reported the highest number of incidents (*n* = 121) followed by Ireland (*n* = 37) and the Netherlands (*n* = 78), while Portugal (*n* = 25), Austria (*n* = 8), Slovakia (*n* = 5), Lithuania (*n* = 2) and Hungary (*n* = 1) followed with a smaller number of incidents. Among all analysed e-cigarette poisoning incidents, 42.7% (*n* = 118) were among children and the remaining 57.2% (*n* = 158) were among adults. There was approximately equal exposure incidents among males and females (50.6% vs. 49.4%). An increase in the number of reported exposures during the reporting period was noted, 10 in 2012, 85 in 2013 and 140 in 2014.

### Exposure characteristics

Unintentional exposure was the most frequently cited exposure (71.3%, *n* = 196), followed by intentional (17.8%, *n* = 49), abuse/misuse (*n* = 21) and suspected suicide (*n* = 3). The distribution of incidents via exposure routes and clinical outcomes is depicted in Table 1. A wide range of symptoms were reported, of which vomiting (20.3%), dizziness (14.5%), nausea (13.8%) and throat conditions (9.1%) were the most frequently reported in both children and adults. Abdominal conditions, eye conditions, headache, diarrhoea, breathing conditions and tremor were also reported. The majority of incidents were managed on site (70.1%), 56 incidents (23.7%) were managed in a hospital and 4 incidents (1.7%) in an ambulance setting.

**Table 1:** Exposure characteristics of reported incidents and most frequently identified clinical effects from European poison Centers, 2012–2015

		Total	
		n	%
Exposure Reason	Unintentional	196	71.3
	Intentional	49	17.8
	Abuse	15	5.5
	Misuse	6	2.2
	Suspected Suicide	3	1.1
	Unknown	6	2.2
Exposure route	Ingestion	187	67.5
	Respiratory	46	16.6
	Dermal	25	9.0
	Ocular	21	7.6
	Other	6	2.2
Initial type of exposure	E-cigarette refill liquid	240	87.3
	E-cigarette non refill	2	0.7
	Unknown type	33	12.0
Clinical effects	Vomiting	56	20.3
	Dizziness	40	14.5
	Nausea	38	13.8
	Throat Conditions	25	9.1
	Throat irritation	9	3.3
	Burning Throat	5	1.8
	Oral Mucosal	8	2.9
	Salivation	2	0.7
	Pharyngitis	1	0.4
	Abdominal Conditions	17	6.2
	Eye Conditions	14	5.0
	Headache	11	4.0
	Diarrhoea	8	2.9
	Breathing Conditions	8	2.9
	Tremor	4	1.4
	Other	75	27.3
Management of incident	Residence/ on site	166	70.1
	Hospital	56	23.7
	Ambulance	4	1.7
	Other/ Unknown	11	4.6
Medical Outcome	Minor effect	112	53.8
	No effect	82	39.4
	Moderate effect	13	6.3
	Major effect	1	0.5
	Death	0	0.0

The majority of recorded exposure incidents had a favourable outcome. In 39.4% of them, no effect was reported and a further 53.8% of the incidents resulted in only minor effects, 6.3% reported moderate effects and 1 case reported a major clinical outcome. No deaths were recorded as a result of an e-cigarette exposure within the data collection period and within our data collected.

As depicted in Table 2, age was associated with the recorded medical outcome, as among incidents which reported clinical effects 65.6% were adults and 34.9% children (*p* = 0.002). Age was also associated with intentional poisoning as the majority of those who reported intentional poisoning using e-liquids were 19 years old or older (*p* < 0.001).

**Table 2:** Bivariate analysis of reported exposures to e-cigarettes from European poison Centers, 2012–2015

	All		≤5 years		6-18 years		Adults (≥19 years)		*p*-value	Intentional		Unintentional		*p*-value	No effect		Effect		*p*-value
	%	n	%	n	%	n	%	n		%	n	%	n		%	n	%	n	
Age																			
5 years	33.2	92	.	.	.	.	.	.		5.5	4	44.4	87	**<0.001**	45.1	37	22.2	28	**0.002**
6-18 years	9.8	27			.	.	.	.		11.0	8	8.7	17		8.5	7	12.7	16	
≥19 years	57.0	158	.	.	.	.	.	.		84.7	61	46.9	92		46.3	38	65.6	82	
Gender																			
Female	49.4	115	50.8	34	37.5	9	52.8	75	0.381	55.9	38	47.8	75	0.264	50.0	39	51.2	63	0.866
Male	50.6	118	49.3	33	62.5	15	47.2	67		44.1	30	52.2	82		50.0	39	48.8	60	
Exposure route																			
Ingestion	67.8	187	87.0	80	59.3	16	57.6	91	**<0.001**	54.8	40	71.9	141	**0.008**	74.4	61	54.8	69	**0.004**
Respiratory	16.3	45	5.4	5	22.2	6	22.2	35	**0.002**	41.1	30	7.1	14	**<0.001**	9.8	8	28.6	36	**0.001**
Dermal	9.1	25	5.4	5	11.1	3	10.8	17	0.339	1.4	1	12.2	24	**0.006**	13.4	11	7.9	10	0.200
Ocular	7.6	21	2.2	2	3.7	1	11.4	18	**0.021**	0.0	0	10.7	21	**0.004**	6.1	5	9.5	12	0.378
Other	2.2	6	2.2	2	7.4	2	1.3	2	0.128	2.7	2	2.0	4	0.730	0.0	0	1.6	2	0.252
Clinical effects																			
Vomiting	20.3	56	20.7	19	29.6	8	18.5	29	0.410	24.7	18	17.9	35	0.212	1.2	1	33.3	42	**<0.001**
Dizziness	14.5	40	7.6	7	25.9	7	16.6	26	0.032	21.9	16	12.2	24	**0.047**	1.2	1	27.8	35	**<0.001**
Nausea	13.8	38	8.7	8	22.2	6	15.4	24	0.139	17.8	13	11.8	23	0.199	1.2	1	28.6	36	**<0.001**
Throat Conditions	9.1	25	6.5	6	18.5	5	8.9	14	0.161	9.6	7	7.1	14	0.506	6.1	5	15.1	19	**0.048**
Abdominal Conditio	6.2	17	5.4	5	7.4	2	6.4	10	0.919	9.6	7	5.1	10	0.179	1.2	1	10.3	13	**0.010**
Eye Conditions	5.1	14	1.1	1	0.0	0	8.3	13	**0.020**	0.0	0	7.1	14	**0.019**	2.4	2	7.1	9	0.139
Headache	4.0	11	0.0	0	11.1	3	5.1	8	**0.019**	6.9	5	3.1	6	0.163	1.2	1	7.9	10	**0.034**
Diarrhoea	2.9	8	0.0	0	7.4	2	3.8	6	0.075	6.9	5	1.5	3	**0.022**	0.0	1	4.8	6	**0.045**
Breathing Conditions	2.9	8	2.2	2	7.4	2	2.6	4	0.334	8.2	6	1.0	2	**0.002**	0.0	0	5.6	7	**0.030**
Tremor	1.4	4	0.0	0	0.0	0	3.2	4	0.215	4.1	3	0.5	1	**0.030**	0.0	0	3.2	4	0.103
Other	27.3	75	20.9	19	33.3	9	29.9	47	0.230	38.4	28	23.1	45	**0.012**	3.7	3	42.9	54	**<0.001**
Clinical outcome																			
No effect	53.8	112	56.9	37	30.4	7	31.7	38	**0.002**	18.4	9	48.3	71	**<0.001**	**.**	**.**	**.**	**.**	
Effect	45.7	96	43.1	28	69.6	16	68.3	82		81.6	40	51.7	76						
Minor effect	39.4	82	38.5	25	52.2	12	62.5	75		81.6	40	51.7	76		**.**	**.**	**.**	**.**	
Moderate/Major effect	6.3	14	4.6	3	17.4	4	5.8	7		0.0	0	0.0	0		**.**	**.**	**.**	**.**	

The numbers in bold are significant with a *p*-value at least <0.05 as defined through Chi-squared tests

Poisoning though ingestion was the most frequent exposure route both for adults and paediatric patients (both those ≤5 years and between 6 and 18 years). Exposure via ingestion was more frequent among paediatric patients (≤5 years) (87.0% vs. 59.3 vs. 57.6 *p* < 0.001) compared with children of 6–18 years and adults, while respiratory exposure was more frequent among adults, and children of 6–18 years of age compared to those ≤5 (22.2% vs. 22.2% vs. 5.4%, *p* = 0.002).

Ingestion was the most frequently recorded route of exposure in both intentional and unintentional incidents (54.8% and 71.9% respectively, *p* = 0.008), while exposure via the respiratory track was the most frequent route of intentional exposure (41.1% vs. 7.1%, *p* < 0.001). Finally, as shown in Table 2, vomiting, dizziness and nausea were the most common clinical symptoms in both intentional and unintentional exposures.

Overall, among incidents that recorded a medical outcome (minor-moderate or major), 54.8% of incidents were associated with ingestion, 28.6% with inhalation, 9.5% of ocular and 7.9% with dermal exposure.

### Regression analysis

Logistic regression analysis assessing factors associated with reporting a clinical outcome indicated that paediatric incidents ≤5 years of age were less likely to lead to clinical effects (aOR 0.41, 95% C.I.: 0.21–0.82) in comparison to older incidents. Gender, intention and type of refill device did not have a significant impact on reporting a clinical effect as outlined in Table 3.

**Table 3:** Parameters associated with the appearance of a clinical effect among e-cigarette exposure incidents reported to European Poison Centers, 2012–2015 (*n* = 172)

		aOR (95% CI)	*p*-value
Age	≥19 years	REF	
	6–18 years	0.80 (0.28–2.27)	0.678
	≤5 years	0.41 (0.21–0.82)	0.011
Gender	Male	REF	
	Female	0.84 (0.44–1.57)	0.580
Intention	Intentional	REF	
	Unintentional	0.25 (0.11–0.59)	0.002
Type	Non-refill/Unknown	REF	
	Refillable	1.04 (0.30–3.61)	0.947

Table 4 depicts the logistic regression analysis that evaluated the factors associated with each route of exposure. Paediatric incidents ≤5 years were 4.36 times more likely to be exposed through ingestion in comparison to adults (95% C.I.: 1.87–10.18) while they were less likely to be exposed through an ocular route (aOR 0.10, 95%C.I: 0.02–0.48). Respiratory exposure was less likely to occur unintentionally (aOR 0.15, 95% C.I.: 0.07–0.32), while dermal exposure was 13.2 times more likely to occur unintentionally (aOR 13.22, 95% C.I.: 1.70–102.56). Refillable e-cigarette liquids were associated more often with exposures via ingestion (aOR 13.05, 95% C.I.:3.11-) and less often via the ocular route (aOR 0.14, 95% C.I.: 0.03–0.71) in comparison to incidents attributed to non-refillable/unknown e-cigarette devices.

**Table 4:** Parameters associated with e-cigarette exposure routes for incidents reported to European Poison Centers, 2012–2105

	Ingestion (*n* = 199)		Respiratory (*n* = 199)		Dermal (*n* = 187)		Ocular (*n* = 156)	
	aOR (95% CI)	*p*-value	aOR (95% CI)	*p*-value	aOR (95% CI)	*p*-value	aOR (95% CI)	*p*-value
Age								
≥19 years	REF		REF		REF		REF	
6–18 years	1.15 (0.45–2.97)	0.766	0.97 (0.30–3.11)	0.958	1.30 (0.32–5.28)	0.709	0.27 (0.03–2.35)	0.236
≤5 years	4.36 (1.87–10.18)	0.001	0.46 (0.15–1.42)	0.176	0.37 (0.11–1.20)	0.096	0.10 (0.02–0.48)	0.004
Gender								
Male	REF		REF		REF		REF	
Female	1.12 (0.62–2.02)	0.717	0.89 (0.42–1.87)	0.596	0.80 (0.31–2.06)	0.648	2.16 (0.78–5.95)	0.137
Exposure								
Intentional	REF		REF		REF		-	-
Unintentional	1.50 (0.79–2.82)	0.215	0.15 (0.07–0.32)	<0.001	13.22 (1.70–102.56)	0.014	-	-
Type								
Non-refillable or Unknown	REF		REF		-	-	REF	
Refillable e-cigarette	13.05 (3.11–54.72)	<0.001	0.32 (0.08–1.27)	0.107	-	-	0.14 (0.03–0.71)	0.017

## Discussion

This is the first study to report exposure incidents attributable to e-cigarettes, as recorded by poison centers in multiple EU MS. Our results indicated that unintentional exposure to e-cigarette refill liquids were responsible for the majority of the reported incidents. The most frequent route of exposure occurred as ingestion or respiration.

The human toxicity of nicotine has become increasingly relevant in the past few years through marketing of new nicotine-containing products, such as smokeless tobacco and liquids for electronic nicotine delivery systems (electronic cigarettes) that are readily available in most countries [12]. This phenomenon has resulted in unprecedented access to potentially toxic doses of nicotine and other harmful compounds in the home [13].

Our results are quite similar in comparison to the US Poison Center report [14]. Vomiting, nausea and dizziness were the most frequent symptoms reported in incidents of e-cigarette exposure both in the US and the EU. Dizziness and throat conditions were more frequent in the EU compared to the US, but the overall profile of clinical effects was quite similar [14]. While one death was recorded in the US dataset, there were no deaths among the European incidents [14].

It is likely that the above symptoms are attributable primarily to nicotine, as historical or experimental evidence has indicated that low doses of nicotine may have stimulant effects (e.g., tachycardia), while vomiting is common following ingestion. Signs of central nervous system toxicity may include ataxia and seizures, while as doses increase, loss of nicotinic receptor specificity may occur and result in signs of muscarinic cholinergic toxicity, including extreme secretions and gastrointestinal disturbance. The highest levels of poisoning can result in neuromuscular blockade, respiratory failure, and death [13]. While the aforementioned symptoms are in line with nicotine poisoning, we cannot rule out that they may also be partially attributable to other ingredients within e-cigarette liquids, which could potentially be harmful if used inappropriately, therefore further research is needed to evaluate the impact of these substances on human health. Our finding that the majority of incidents reported in poison centers refer to unintentional exposure is also consistent with published data [14]. However, several incidents of intentional poisoning –suicide attempts- through ingestion have also been reported in the literature [15–22].

Our results also indicated that paediatric patients are more likely to be exposed through ingestion of e-cigarette liquids, which is a subject of concern. This finding is in line with data from the UK National Poisons Information Service, which indicated that 36.4% of the exposure incidents were for children younger than 4 years old [17] while a CDC report noted that young children accounted for more than 50% of the incidents [4].

Our results also indicated an increase in the number of exposure incidents during the study period, a factor which we hypothesize is associated with the increase in popularity, and hence availability, of e-cigarettes and their refill vials in Europe [11]. Nevertheless, the number of incidents reported to national poison centers were not proportional to either the country’s population or the prevalence of e-cigarette use. Therefore, they might also reflect reporting procedures or differences in the products used in each country [11]. Similarly, events related to e-cigarette product exposure reported to the American Association of Poison Control Centers increased from 2011 to 2014 [23–25]. A recent retrospective analysis of exposures associated with nicotine and tobacco products among children younger than 6 years old conducted using US National Poison Data System data also indicated a continuing increase in the number of exposures associated with e-cigarettes, which increased almost 15-fold between 2012 and 2015. The increase in the number of exposure incidents and the identified age specific epidemiology of exposures highlights the need for the implementation of design features that could mitigate the incidence of e-cigarette poisonings [26].

Within the EU, the TPD implementing acts stipulate the need to incorporate child and tamper resistant caps, a parameter that may reduce paediatric incidents attributable to ingestion, while Article 20(3)(g) of the TPD requires EU Member States to ensure that electronic cigarettes and their refill containers have a mechanism that ensures refilling without leakage, a fact that would mitigate the likelihood of dermal/ocular exposures to users. Our study was completed before the implementation of such provisions, therefore further prospective studies are needed so as to assess if the proposed technical parameters would impact the epidemiological trends and characteristics of exposure incidents in EU MS.

This is the first study to report exposure incidents recorded by poison centers in multiple EU MS, results of which are imperatively needed so as to map the existing status quo of e-cigarette exposure incidents before the implementation of the regulations of the TPD in 2016. Despite the above, our analysis has limitations as results cannot be generalized beyond the ten EU MS that contributed data in this analysis, however the alignment with findings from US poison centers indicate the potential common epidemiology of exposure incidents attributable to e-cigarettes. Furthermore as data was retrospectively collected and de-identified and hence limited to those obtained from archive records, further in depth analysis of the data were restricted due to the small sample size of a number of exposure characteristics which led in some cases to wide confidence intervals. Further research using larger datasets is warranted.

## Conclusions

Our study highlighted specific exposure characteristics and routes associated with involuntary exposure to e-cigarette refill liquids, with pediatric incidents more likely to be attributable to ingestion. These results indicate that consideration should be given in the technical design features of e-cigarette refill vials, which may limit the risk of accidental ingestion and risks associated with the refilling processes and thereby protect both users and non-users, and especially children. Further research is needed after the implementation of the legislative design features outlined in the TPD so as to assess their impact on the incidence and characteristics of future exposure incidents.

## Abbreviations

ENDS: Electronic Nicotine Delivery Systems
EU DG Health and Food Safety: European Directorate General for Health and Food Safety
EU MS: European Member States
FDA: Food and Drug Administration
TPD: Tobacco Products Directive
UK: United Kingdom
US: United States
